# Pathology in General Practice

**Published:** 1907-04-13

**Authors:** 


### PATHOLOGY IN GENERAL PRACTICE.

### The Demonstration of the Different Cocci and their Significance.

The different forms of cocci may all be very easily stained by any of the ordinary basic aniline dyes. For the general practitioner who is not dealing with cultures, the organisms will have to be detected in pus, sputum, urine, and other discharges, and he must proceed by making smears, of whichever of these he is to examine, on slides or coverslips. It is essential that such smears should not be too thick, and the best way of making them is to use a looped platinum needle to spread out the material. After this they must be fixed, and this is easily accomplished by passing them several times over the flame of a Bunsen burner, a very useful practical point being to hold the slip or slide in one's fingers so as to be able to tell when too much heat is being applied. All this having been satisfactorily accomplished, a stain has next to be selected, and it is convenient to have a series of stock solutions ready prepared on one's bench, one, say, of methylene blue, one of thionin blue, and one of fuchsin. These ought to be kept in ordinary glass bottles that have had their stoppers replaced by glass funnels with filter paper, so that each time before use all that will be required is to drop a little of the fluid into the funnel, and, holding this over the slide, to let the filtered result drop on the film. This ensures a clean specimen, and no deposit of stain to obscure and mask the picture. The following are the formulæ of some of the commoner stains:—

I. *Methylene Blue Saturated Aqueous Solution,*

or

II. *Methylene Blue Saturated Alcoholic Solution.*

Stain for half a minute, wash, dry, and mount.

III. *Alkaline Methylene Blue* (Löffler's solution).

| | |
|---|---|
| Saturated solution of methylene blue | 30 parts |
| Solution of caustic potash of 1 : 10,000 | 100 parts |

Stain for half to one minute. If colour too deep, decolorise with ½ to 1 per cent. acetic acid till film becomes pale-blue green, wash, dry, and mount.

IV. *Kühne's Methylene Blue.*

| | |
|---|---|
| Methylene blue ... ... ... | 1.5 parts |
| Absolute alcohol ... ... ... | 10 parts |
| Carbolic acid (5 per cent.) ... ... | 100 parts |

Stain for ½ to 1 minute or longer, decolorise if necessary with weak acetic or hydrochloric acid. Wash, etc.

V. *Thionin Stains.*

| | | |
|---|---|---|
| *a.* | Thionin crystals ... ... ... | 1.5 parts |
| | Absolute alcohol ... ... ... | 10 parts |
| | Carbolic acid (5 per cent.) ... ... | 100 parts |
| | or | |
| *b.* | Thionin crystals ... ... ... | 1 part |

Dissolve in 100 parts carbolic acid solution 1-40, dilute one volume with three of water, and filter; stain for ½ to 1 minute with the stronger. If decolorisation is required acetic acid may be employed. Wash, etc.

VI. *Carbol Fuchsin Stain.*

| | |
|---|---|
| Fuchsin ... ... ... ... | 1 part |
| Absolute alcohol ... ... ... | 10 parts |

Dissolve and add 100 parts of a 5 per cent. solution of carbolic. Stain for half a minute for ordinary films.

Besides those there are plenty of others, the more important being methyl violet, gentian violet, toluidin blue, Victoria blue, safranin, Bismarck brown, etc. After staining, all films should be very thoroughly washed, then dried and examined with the $\frac{1}{12}$ in. oil immersion lens, direct if only required for diagnosis, mounted in Canada balsam if for permanencies. There is absolutely no difficulty, then, in this technique, and for future reference we will refer to it as simple staining. The next method is more complicated, but is important because by it some of the different cocci can be differentiated from each other: it is termed Gram's stain or Gram's method. For this we require gentian violet, aniline oil, a solution of iodine, alcohol, and some counter-stain, preferably a pink one. The aniline oil and gentian violet are mixed together as follows:—Take a test tube and fill it with distilled water; to this add a few drops of aniline oil, and shake it up until an emulsion is formed; then filter this into a small glass flask. A certain amount of the oil, it will be found, has dissolved in the water, and this may therefore be termed aniline water. Next, to this add a few drops of a saturated alcoholic solution of gentian violet, enough to make the solution deep purple, and then we have our aniline gentian violet. The iodine solution consists of iodine 1 part, potassium iodide 2 parts, distilled water 300 parts. There are different ways of using the stain, some preferring counter-staining first, others later; but the following may be taken, not specially as the best method, but as useful as any. The film, say of pus, sputum, or other discharge, having been made in the usual manner and fixed, apply a little aqueous or alcoholic solution of eosin to it for half a minute. Wash. Pour on aniline gentian violet for five to ten minutes, washing this off in turn with water. Iodine solution then for one minute. After this, alcohol till the violet colour all disappears and the original pink colour returns; then dry, and mount. If Gram staining cocci are present, and the method has been properly carried out as described, the organisms will be purple, the tissues pink, the contrast being very striking. Most of the cocci produce pus, and those that chiefly concern us are the staphylococci, the streptococci, the pneumococcus, the gonococcus, and the meningococcus. The former, the staphylococci, get their name by being shaped like clusters of grapes; they are widely distributed in nature, being present in the dust of rooms, the mouth, the skin, and other places; while in pathological conditions, such as acne spots, boils, abscesses, and the like, they are usually present and are, indeed, the causative agent. They can easily be demonstrated by making smears on slides, staining by any of the simple methods, or by Gram's stain, as they retain the latter. The different species cannot, however, be detected morphologically by staining; for this we require to grow them on different culture media, where they develop and produce different colours and features. In our next issue we hope to be able to discuss the streptococci, gonococci, pneumococci, and others of importance.

